# Decreased Cytotoxicity of Peripheral and Peritoneal Natural Killer Cell in Endometriosis

**DOI:** 10.1155/2016/2916070

**Published:** 2016-05-12

**Authors:** InCheul Jeung, Keunyoung Cheon, Mee-Ran Kim

**Affiliations:** ^1^Department of Obstetrics and Gynecology, College of Medicine, The Catholic University of Korea, 64 Daeheung-ro, Jung-gu, Daejeon 34943, Republic of Korea; ^2^Department of Obstetrics and Gynecology, College of Medicine, The Catholic University of Korea, 59 Dongsu-ro, Bupyeong-gu, Incheon 21431, Republic of Korea; ^3^Department of Obstetrics and Gynecology, College of Medicine, The Catholic University of Korea, 222 Banpodae-ro, Seocho-gu, Seoul 06591, Republic of Korea

## Abstract

Endometriosis causes significant chronic pelvic pain, dysmenorrhea, and infertility and affects 10% of all women. In endometriosis, ectopic endometrium surviving after retrograde menstruation exhibits an abnormal immune response characterized by increased levels of activated macrophages and inflammatory cytokines. Particularly, dysfunctional natural killer (NK) cells play an important role in the pathogenesis of the disease by either facilitating or inhibiting the survival, implantation, and proliferation of endometrial cells. NK cells in the peritoneum and peritoneal fluid exhibit reduced levels of cytotoxicity in women with endometriosis. Several cytokines and inhibitory factors in the serum and peritoneal fluid also dysregulate NK cell cytotoxicity. Additionally, increased numbers of immature peripheral NK cells and induction of NK cell apoptosis are evident in the peritoneal fluid of women with endometriosis. The high rate of endometriosis recurrence after pharmaceutical or surgical treatment, which is associated with dysfunctional NK cells, indicates that new immunomodulatory management strategies are required. A good understanding of immune dysfunction would enable improvement of current treatments for endometriosis.

## 1. Introduction

In endometriosis, ectopic endometrium survives, causing a disease characterized by implantation of endometrial tissue outside the uterus; this, in turn, triggers pain and infertility. Ectopic endometrium, which is thought to originate via retrograde menstruation, causes significant chronic pelvic pain, dysmenorrhea, and infertility, accompanied by inflammatory changes [1, 2]. This widespread estrogen-dependent disease is estimated to affect 10–15% of all women and up to 50% of women with chronic pelvic pain and infertility [3–5]. Almost 50% of adolescents with intractable dysmenorrhea or pelvic pain are diagnosed with endometriosis, but it is not yet clear why only certain women develop the condition [3].

The most widely accepted theory, which was developed by Sampson, holds that that endometrial tissue refluxed to the Fallopian tubes fails to be cleared and attaches to the peritoneum. Some 70% of women who menstruate regularly exhibit bleeding reflux, but only 10% develop endometriosis [6–8]. Several factors are likely to influence susceptibility to the condition. The high rate of recurrence of endometriosis after pharmaceutical or surgical treatment indicates that researchers need to further define the pathophysiology of the condition, which, in turn, would facilitate work toward an effective treatment.

Recently, it has been suggested that abnormal immune function and dysregulation of immune mediators are responsible for the poor response to treatment, and poor clearance, of ectopic endometrium. Immune status is now considered to play an important role in the initiation and progression of endometriosis. Several studies have shown that the levels of activated macrophages, T cells, B cells, and inflammatory cytokines are increased in women with endometriosis [9, 10]. Specifically, natural killer (NK) cells have been suggested to play an important role in the pathogenesis of the disease by either allowing or inhibiting the survival, implantation, and proliferation of endometrial cells [11, 12]. Reductions in NK cell cytotoxic function have been observed in the peritoneal fluid (PF) of patients with endometriosis [13, 14], implying that a defect in NK cell cytotoxic function, preventing elimination of endometrial cells from ectopic sites, may cause endometriosis.

In this review we define the immunological changes evident in women with endometriosis, with a specific focus on NK cells and the contributions of immunological factors to reductions in the functions of such cells.

## 2. Role of the Immune System in Endometriosis

Immune cells play key roles in the detection and clearance of abnormal cells [15]. It has been proposed that impairment of the immune response, resulting in inadequate removal of refluxed menstrual debris, is an important contributor to endometriosis [16, 17]. Recent studies on the immunological changes associated with endometriosis have focused on the significance of NK cells.

## 3. Cellular Immunological Changes in the Peritoneal Cavity of Women with Endometriosis

Endometrial fragments refluxed during menstruation induce inflammation within the peritoneal cavity [18]. Normally, neutrophils and macrophages are among the first immune cells to be recruited to this area. Macrophage numbers are increased in the PF of patients with endometriosis [19]; however, these cells fail to act as scavengers of endometrial tissue and are primary contributors to the elevations in proinflammatory and chemotactic cytokine levels found in the PF [20]. In addition to encouraging the growth of peritoneal implants, macrophages are a major source of angiogenic mediators, including TNF-*α* and IL-8 [21]. Macrophages seem to be involved in the growth and development of endometriotic tissue, but macrophage depletion does not prevent endometrial cell implantation in the peritoneum, suggesting that the mechanisms of implantation and pathogenesis differ [22, 23].

The neutrophils of women with endometriosis exhibited a slower rate of apoptosis than did those of control women [24]. Dendritic cells (DCs), a type of antigen-presenting cells (APC), activate naive T cells to become cytotoxic or T helper cells. One study found that depletion of DCs caused growth of an endometriotic lesion [25], but another reported that DC depletion attenuated the development of endometriosis [26]. The role played by DCs thus needs further study.

## 4. Natural Killer Cells

NK cells, which comprise 15% of all circulating lymphocytes, particularly those of the innate immune system, protect against tumor development and viral infections. The cells have both cytolytic and immunomodulatory capabilities [27, 28]. NK cells destroy other cells by secreting lytic granules containing granzymes, perforin (at immune synapses), and cytotoxins or cytokines, such as IFN-*γ* [29, 30]. Significant populations of NK cells are found in lymphoid tissues, such as the bone marrow and blood, as well as in nonlymphoid tissues, such as the liver and gut [31–33].

The level of CD56 expressed by NK cells appears to correlate with NK cell function: CD56dim NK cells are more cytotoxic and express higher levels of immunoglobulin-like NK cell receptors and FC-*γ* receptor III (CD16) than do CD56bright NK cells. In contrast, CD56bright NK cells are potent producers of cytokines, particularly IFN-*γ* and TNF-*α*, following activation by monocytes, but they exhibit low-level natural cytotoxicity and low levels (or an absence) of the FC-*γ* receptor CD16.

## 5. NK Cell Dysfunction and Endometriosis

Following translocation of refluxed endometrial tissue into the peritoneal cavity, the endometrial fragments must survive the immune response and attach and invade the peritoneal membrane to establish a lesion. In endometriosis, dysfunctional NK cell cytotoxicity may allow endometrial fragments to survive in the peritoneum [34].

Most studies have found that the numbers of cytotoxic NK cells are reduced in the PF and peripheral blood of endometriosis patients and that this is accompanied by an overall decrease in NK cell activity [14, 35–37]. In such patients, the populations of NK cells (CD32CD56+) are significantly decreased, whereas the proportions of immature NK cells (CD272CD11b2) among CD32CD56+ NK cells are increased in the PF. Functional impairment and diminished cytotoxicity of NK cells within the peritoneal cavity have also been well documented in such patients [34]. The NK cell levels of granzyme B, perforin, TRAIL, and CD107a are reduced in the PF of patients with endometriosis, indicating that the NK cells are functionally defective.

The levels of the inflammatory cytokines IL-6, IL-8, IL-1b, IFN-*γ*, and TNF-*α* increase in the PF of patients with endometriosis, which is consistent with the elevated levels noted in the serum [38–41]. Certain chemokines, especially CXCL8 (IL-8), CCL-2 (MCP-1), and CCL5 (RANTES), can serve as biomarkers identifying patients with endometriosis, but the accuracy of such tests can be improved by including other noninflammatory markers in the biomarker panel.

## 6. An Altered NK Cell Phenotype in the Peritoneal Cavity

Markers of NK cell cytotoxicity include the natural receptors NKp46, NKp44, and NKG2D, CD16 (a cell surface marker), and CD107a [42] and CD69 (markers of activation). The levels of all of these markers are significantly reduced in the peritoneal NK cells of endometriosis patients. One study found that NK cell cytotoxicity was reduced in the PF of endometriosis patients but recovered upon immunomodulatory treatment; the expression levels of the activation marker CD107a were compared before and after treatment.

The levels of most cell-activating receptors are decreased when NK cells are downregulated, whereas the levels of most inhibitory receptors are increased upon upregulation. Such up- or downregulation may be mediated by cytokines, but the detailed mechanisms remain unknown.

One study found that expression of the human leukocyte antigen-G (HLA-G; the ligand of KIR2DL4) in ectopic endometrial cells prevented detection of such cells by patrolling NK cells, allowing survival of the endometrial cells and implantation of the peritoneum [43]. The level of the inhibitory cytotoxic receptor for HLA-E, CD94/NKG2A, was also significantly increased on the peritoneal NK cells of endometriosis patients; the receptors may inhibit the release of cytolytic granules by such cells, allowing endometriotic lesions to grow [44, 45].

Killer cell inhibitory receptors (KIRs) are representative receptors recognizing major histocompatibility complex class I molecules on target cells; the receptors regulate NK cell cytotoxicity to target cells. Such inhibitory NK cell receptors contain Ig domains (KIR2DL1, KIR2DL2, KIR3DL1, and KIR3DL2) in their extracellular regions [46] and immunoreceptor tyrosine-based inhibitory motifs (ITIMs) in their cytoplasmic portions [47, 48]. Ligand binding facilitates the recruitment of SHP-1 and SHP-2 and the suppression of immune responses, including NK cell cytotoxicity [49, 50]. Many studies have reported upregulated levels of ITIM-KIRs, KIR2DL1 [51–53], NKB1, EB6 [54], the soluble intracellular adhesion molecule-1 (I-CAM), and HLA-I in the PF of endometriosis patients; the levels of cytokines correlated directly with the extent of inhibition of NK cytotoxicity [55, 56].

Recently, IL-6 in the PF of endometriosis patients has been identified as a possible immunosuppressant of NK cell cytotoxicity, and it may play a crucial role in impairing NK cell function by regulating SHP-2 expression [47].

Women with endometriosis have higher numbers of immature peripheral NK cells than do those without the disease [36]. The proportion of mature NK cells increases after surgical removal of endometrial lesions, suggesting that certain cytokines produced by the lesions influence the differentiation of peripheral NK cells.

## 7. Cytokines in the PF of Endometriosis Patients

The TNF-*α* level is increased in the PF of women with endometriosis [57–59], but clinical trials of anti-TNF-*α* therapies did not alleviate pain symptoms [60].

The level of FasL, which induces NK cell apoptosis by binding to the ligand receptor CD95, was increased in the PF of women with endometriosis [61, 62], and endometriosis peritoneal NK cells expressed significantly elevated levels of CD95 [63]. Thus, NK cells in the PF may undergo FasL-induced apoptosis, allowing the endometrial cells to survive.

The level of IL-6, another inhibitory cytokine, is dramatically increased in the PF of patients with endometriosis. The levels of mRNAs encoding IL-6-upregulated TFs, c-Myc, and SOCS-3 are also increased in PF cells, and IL-6 signaling has been shown to regulate cell growth, differentiation, and survival. Furthermore, IL-6 directly affects the differentiation of cytotoxic NK cells from CD34+ cells. IL-6 also affects the functional activities of such cells by increasing the levels of mRNAs encoding SHP-1 and SHP-2. IL-6 also increases KIR expression on NK cells (CD56+ cells) [64]. Some study shows that NF-*κ*B may be one of major culprits in the pathogenesis of endometriosis, and its constitutive and inducible activation may be responsible for antiapoptosis, angiogenesis, invasiveness, and increased production of proinflammatory cytokines and chemokines [65].

## 8. Conclusions

The immunoregulatory factors involved in the NK cell cytotoxicity in PF and peripheral blood of women with endometriosis are shown on Table 1. The pathogenesis of endometriosis is associated with abnormal differentiation and function of cytotoxic NK cells. The phenotypes of peripheral blood and PF NK cells in women with endometriosis indicate that the NK cells are dysfunctional. Specifically, NK cells of the peritoneum and PF are less cytotoxic in women with endometriosis than in control women. Several cytokines and inhibitory factors in the serum and PS also negatively affect NK cell cytotoxicity. It remains possible, however, that the NK cell abnormalities evident in women with endometriosis are, in fact, consequences of the pathology. Future research should seek to explain how the observed immunological changes in, and dysfunctionality of, NK cells are initiated. Novel strategies to manage endometriosis should be based on an understanding of the associated immunological problems and should prioritize improvements in NK cell functionality.

## Figures and Tables

**Table 1:** Change of the immunoregulatory factors in the NK cell cytotoxicity.

Decreased cytotoxic activity	Increased inhibitory activity
Cytotoxic function	Inflammatory cytokines
Granzyme B, perforin, TRAIL, CD107a	IL-6, IL-8, IL-1b, IFN-*γ*, TNF-*α*
Cell-activating receptors	Noninflammatory cytokines
NKp46, NKp44, NKG2D, CD16 (cell surface marker)	CXCL8, CCL-2 (MCP-1), CCL5 (RANTES)
CD69 (markers of activation)	Antigen
	HLA-G, HLA-E, HLA-I
	Inhibitory receptors
	ITIM-KIRs, KIR2DL1, NKB1, EB6, I-CAM
	Apoptosis
	FasL (CD95)
